# In Situ Detection of Water Leakage for Textile-Reinforced Composites

**DOI:** 10.3390/s20226641

**Published:** 2020-11-20

**Authors:** Julie Regnier, Aurélie Cayla, Christine Campagne, Eric Devaux

**Affiliations:** Ecole Nationale Supérieure des Arts et Industries Textiles (ENSAIT), Institute Centrale Lille, GEMTEX, Laboratoire de Génie et Matériaux Textiles, 2 Allée Louise et Victor Champier, 59056 Roubaix CEDEX 1, F-59000 Lille, France; julie.regnier@ensait.fr (J.R.); christine.campagne@ensait.fr (C.C.); eric.devaux@ensait.fr (E.D.)

**Keywords:** water leak detection, composite resin, metallic and carbon-based conductive yarns

## Abstract

By incorporating electrically conductive yarns into a waterproof membrane, one can detect epoxy resin cracking or liquid leakage. Therefore, this study examined the electrical conductivity variations of several yarns (metallic or carbon-based) for cracking and water detection. The first observations concerned the detectors’ feasibility by investigating their conductivity variations during both their resin implementation processes and their resin cracking. Throughout this experiment, two phenomena were detected: the compression and the separation of the fibres by the resin. In addition, the resin cracking had an important role in decreasing the yarns’ conductivity. The second part of this study concerned water detection. Two principles were established and implemented, first with yarns and then with yarns incorporated into the resin. First, the principle of absorption was based on the conductivity variation with the yarns’ swelling after contact with water. A short circuit was established by the creation of a conductive path when a drop of water was deposited between two conductive, parallel yarns. Through the influence of the yarns’ properties, this study explored the metallic yarns’ capacity to better detect water with a short circuit and the ability of the carbon-based yarns to detect water by the principle of absorption.

## 1. Introduction

Over the last decade, the development of new detectors for the environmental, medical, scientific and athletic fields has been increasing. These detectors are used to control physical parameters. They are only acquisition systems and require storage or display systems to process the data. Thus, detectors are used to change an observed physical or chemical quantity into a usable signal. For instance, they are able to detect a variation in temperature, stress or displacement and transform it into electric tension. The signal can be logical (with two states), digital (discrete values) or analogical (usually a voltage or electrical current signal). In the environmental field, one of their applications is infrastructure monitoring, such as in the case of a liquid leak. For this function, the detector can be incorporated in composites, for example, a textile-reinforced concrete (TRC) structure. These composites contain a textile, which can be fabric, knitting [1] or multiaxial and is usually composed of glass fibres due to their strong resistance properties [2]. The reinforced textile is then moulded into a resin or concrete. For instance, in storage infrastructures, extreme strain and thermal stress caused by environmental ageing (e.g., thermic variation and moisture) are applied to the composite and can cause a crack [3] or liquid leak.

On this note, there are different types of fluid leakage detectors. They can be based on different technologies, such as thermal, mechanical or chemical detection. One of them is the optical fibre, which is based on a thermal variation and mechanical deformation and modifies the propagation of the light ray inside the fibre [4]. This technology can be suitable in the infrastructure-monitoring field, especially for the monitoring of fluid leaks in a pipeline. Such a system can detect over a long distance, but its installation is difficult due to its dependence on seasonal temperature variations and other environmental factors. If the structure is damaged, the detector with optical fibres can be damaged as well. Furthermore, replacing the latter is uneconomical and decreases the quality of detection [5].

Most of the detectors used for fluid leakage are based on an electrical signal technology [6], such as liquid leak detection cables, which are mostly used for pipeline monitoring [7]. These detectors are based on the chemical properties of the relevant liquid or gas and modify the electrical conductivity of their components (electrically conductive yarns). For leak detection on the pipeline, the detection cables are made with different components: a braid of polyester yarns and an electrically insulated silicone detection layer, which are used to protect the metallic detection yarn [8]. This detection system can locate a fluid leak but only on a pipeline by running the detection cable under the pipeline. It cannot locate a fluid leak inside a storage structure. In the clothing industry, conductive inks also employ electrical signal technology for liquid detection using inorganic components (with metallic particles) or, more often, organic components (with carbon particles) [9]. This application is expensive and more suitable for small, printed circuits than large structures.

To detect larger structures using less expensive materials, the principle of the electrical conductivity’s variation can be used with intrinsically conducting polymers (ICPs) [10,11,12] or conductive polymer composites (CPCs) [13,14,15]. CPCs are formed from a matrix of polymers and a sufficient quantity of fillers to make the yarn conductive. These two electrical materials, based on the variations in their conductivities, detect liquid or a mechanical deformation. They can be found in the forms of a film [16], a monofilament [17] or a multifilament [18] or incorporated into a textile [19]. The advantage of using a conductive material (CPC or ICP) to detect a fluid is that its structure is modified according to the affinity of the polymer with the fluid [20,21]. If there is a strong affinity between them, the fluid diffuses inside the polymer, resulting in a swelling of the matrix. This phenomenon induces a modification of the distance between the conductive path inside the yarn and, consequently, a variation in its electrical conductivity. This property of absorption to detect liquids or fluids is not yet commonly used in industry but is more at the research stage in the relevant literature.

On this subject, some researchers have used metallic yarns in a textile [22,23] to detect fluid on a larger textile structure. Xu et al. have worked on a water detector in textiles [22]. They used the principle of the short circuit, which is based on two conductive yarns in parallel without initial contact. The conductive yarns are woven, knitted or embroidered onto a non-conductive yarn support. To permit contact between both and activate the electrical conductivity of the circuit, the water must create a connection between the two conductive yarns on the textile. Often, in cotton or polyester, the non-conductive yarns absorb and spread water on the textile to create the conductive path between the two conductive yarns and the water. Three different conductive yarns have been studied with this principle: stainless steel (SS) yarns, SS blended with polyester yarns and silver-plated multifilaments. The most suitable yarns are the silver-plated multifilaments because the resistance of the stainless steel-blended yarns is too high (up to hundreds of kΩ/cm) due to the dispersion of the stainless steel fibres in the blend. This study was based on the principle of a short circuit with a metallic yarn on a textile support, but it has not been investigated in a resin.

To monitor the behaviour of large infrastructures and detect fluid leakages in the waterproof membrane of concrete, some studies have been conducted by incorporating smart textiles into the structures. Infrastructures are generally composed of different layers, including a membrane, which compensates for the ductile property of the concrete [24]. This waterproof membrane is a composite of a reinforced-fibre textile and a resin. Adding conductive yarns to the textile improves its functions in the composite by adding the function of monitoring the behaviours of the membrane. Goldfeld et al. already explored water leak detection in a concrete membrane with conductive yarns in carbon [25] or metallic [26]. They studied the electrical conductivity variation of the yarn in a concrete plate during the cracking process. In addition, they observed different principles of water leak detection. The first one was the principle of the conductivity’s variation. The electrically conductive yarn uses its property of absorption to detect the water and modify its conductivity to transmit the information. The second principle was the short circuit. The study showed that the water could be easily detected by the short circuit with metallic yarns, but this configuration deviated from the detection of strain deformation. This research also showed that the principle of detection by the yarn’s property of absorption is more adapted for carbon yarns than for metallic yarns. Thus, the variation of the electrical conductivity within the carbon yarn was more relevant (about 10^−5^ Ω).

In our study, different types of conductive yarns were investigated in an epoxy resin. This panel of conductive yarns included stainless steel (SS)-based yarns and CPC yarns. Various properties of these conductive yarns were investigated through two water-detection principles: water detection by absorption and by short circuits. The signal of the short circuit additionally depended on several parameters: the properties of the liquid (the chemical nature, ionic conductivity or surface tension) and of the two conductive yarns (the distance between them and the textile structures). The different yarns’ properties investigated with the water detection principles were the physical structures of two SS yarns: a spun yarn and multifilament; the hydrophilic properties of the non-conductive fibres; and the proportion of SS in a spun yarn and the formulation of three CPCs. The incorporation of these yarns into the resin was studied to examine the resin’s influence on the yarns’ conductivity variations. To validate the hypotheses of the resin’s influence, certain scanning electron microscope images of yarns implanted in the resin were utilized. Next, a crack creation with three-point bending was realized to investigate the crack’s influence on the yarns’ conductivity variation. The influence of all of the yarns’ properties on the water detection was tested without resin first and later in the cracked resin.

## 2. Materials and Methods

### 2.1. Materials

#### 2.1.1. Conductive Yarns

To observe the influence of certain properties on water detection, several electrically conductive yarns were tested in this study. First, SS-blended spun yarns were used to investigate the hydrophilic property of the non-conductive fibres in the blend. The SS fibres could be blended with fibres of polyacrylate (PAC), viscose (VIS) or polyethylene terephthalate (PET). The blended spun yarns also permitted the observation of the influence of the SS proportion on the SS/PET blend (Table 1). All of the spun yarns were supplied by the IMATTEC Company, except the SS60/PET40 yarn, which was supplied by CREAFIBRES. The second category included two SS yarns with different physical structures: a multifilament or spun yarn (Table 2). The last category of electrically conductive yarns included three different formulations of conductive polymer composites (CPC) (Table 3). One was a multifilament obtained by melt spinning and made of polyamide 12 (PA12), incorporated through extrusion with carbon filler materials: carbon nanotubes (CNTs) and carbon blacks (CB). The other yarn was a monofilament, a blend of polypropylene (PP) and polycaprolaptone (PCL). It was formed by two successive extrusions. The first one aimed to incorporate and disperse the carbon nanotubes in the PCL, and the second one permitted the biphasic blend of the PCL_CNT_ with the PP. Next, this blend was used to coat an SS yarn through the extrusion process. Finally, the last formulation was a monofilament obtained through two successive extrusions [27]: with a low-density polyethylene (PE) with carbon fillers that were CNTs and a highly structured carbon black called Ketjenblack (KB) and then with a propylene-based elastomer (PBE).

#### 2.1.2. Distilled Water

To reduce water-related uncertainties and make the protocols repeatable, the quality of the distilled water was controlled before each test. Its surface tension, measured by the tensiometer “3S Scales” from GBX Instruments, is about 71.8 mN/m, with a standard deviation of 3.2 mN/m. Since the detection’s mechanisms depend directly on the ionic conductivity of the water [26], this parameter was controlled with a conductimeter (Tacussel electronic type cdrv 62) and a conductivity standard solution of 1413 μS/cm at a temperature of 25 °C (KCL, Fischer Scientific). The conductivity value of the distilled water was 0.1 ± 0.07 μS/cm.

#### 2.1.3. Resin

The waterproof resin was composed of an epoxy base with pigments in synthetic oxides and an amine hardener. The base and the curing agent amine component were mixed in a glass beaker at room temperature in proportions by weight of base 3/hardener 1. The resin was dried out in a mould for eight hours at room temperature.

#### 2.1.4. Design and Production of Sample: Implementation

**Figure 1:**
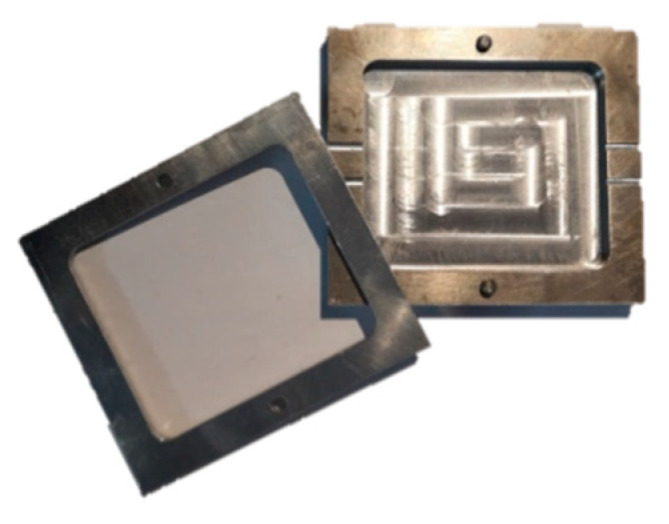
Mould used to form the composite.

**Figure 2:**
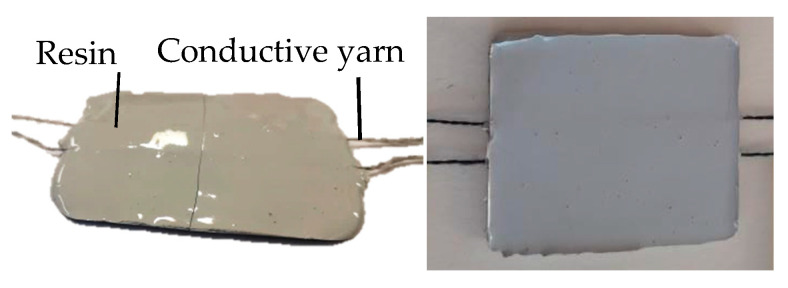
Example of the conductive yarn resin in the mould (side and front view).

To imitate the detection of water leakage in a waterproof membrane, the yarns were implemented in an epoxy resin, and then, the samples were cracked owing to three-point bending. Five samples were made for each yarn. In order to form the samples, the resin was cast in the inferior part of a mould (Figure 1), the conductive yarns were stretched on this inferior part and the superior part was added to cast the resin above the yarns (Figure 2). All of the samples measured 70 mm long, 60 mm wide and 3 mm thick.

**Figure 3:**
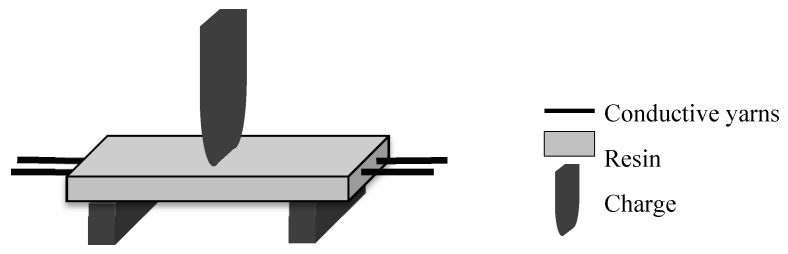
Three-point bending test scheme.

The crack in the middle of the resin was realized with the three-point bending method (Figure 3) on a universal testing machine (model 5985, force capacity: 250 KN, INSTRON, Elancourt, France) with software called Bluehill. The parameters were chosen to create a crack in the resin while avoiding breaking the conductive yarns inside the sample. The tests were carried out at a quasi-static speed of 0.25 mm/min with a load threshold at 100 N.

### 2.2. Methods of Characterization

To examine the influence on the water detection, all of the yarns and the monofilaments were first studied without the resin by controlling their electrical conductivity according to the two principles of detection: absorption and short circuits. Their implementation in the resin and the cracking method were examined through their electrical conductivity’s variation. In addition, the scanning electron images allowed the observation of the yarns’ incorporation into the resin.

#### 2.2.1. Scanning Electron Microscope (SEM)

Scanning electron microscopy (SEM) was performed with a SEM-FEG Scios2 scanning microscope. First, the samples were prepared by cutting them to the desired thickness with a sectioning saw (IsoMet Low Speed Saw, BUEHLER, Esslingen am Neckar, Germany). Next, they underwent an ionic polishing and a metallization with platinum before being observed under a microscope using charge-contrast imaging at 10 kV.

#### 2.2.2. Measure of the Electrical Conductivity

All of the water detection tests to measure the electrical conductivity were realized five times for each yarn and conditioned at a room temperature of 20 °C and a humidity of 65% to validate the results and the repeatability of the protocols.

For all of the electrical conductivity tests, the yarns were connected to an ohmmeter (TTi computing multimeter model 1906, 5½ Digit, Aim-THURLBY THANDAR INSTRUMENTS, Huntingdon, UK), which allowed a continuous configuration. First, the initial resistance of all the dry yarns was measured. Each yarn was attached by two alligator clips, which were separated by a distance of 10 cm and connected to the ohmmeter. Next, their electrical conductivity was calculated with Equation (1):

(1) $$\sigma \;\left(S/m\right)=\frac{L}{RS}$$

where *σ* is the electrical conductivity of the system, *L* is the yarn’s length (*L* = 0.1 m), *S* is the yarn’s area (m^2^) and *R* is the resistance measured (Ω).

**Figure 4:**
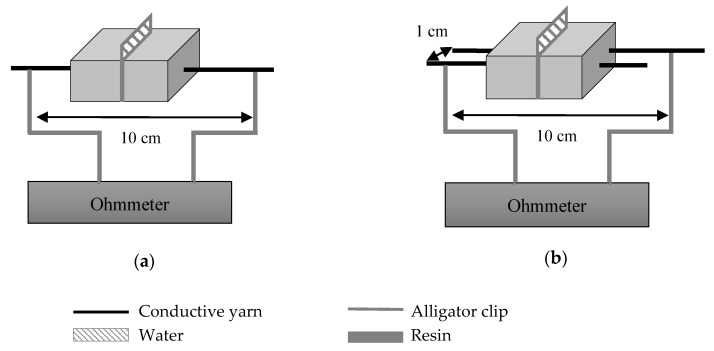
Principle of water detection by absorption (**a**) and by a short circuit (**b**) with the electrically conductive yarns incorporated into a resin plate, and the same schemes—but without resin—for the study of detection principles on conductive yarns only.

The variation of the electrical conductivity of the yarns was measured at different steps of their implementation into the resin membrane (Table 4). First, the principle of detection by absorption was studied (Figure 4a, without resin). This detection system was based on the modification of the initial resistance of the yarn when it was in contact with a drop of water. Using this value, the electrical conductivities of the dry and wetted yarn were calculated (Equation (1)). To observe the influence of the yarns’ properties on the detection, the detector sensitivity (*Sw*) calculated corresponded to the rate of change in the electrical conductivity between the dry yarn and the wetted yarn (Equation (2)).

(2) $$Sw\;\left(\%\right)=\;\frac{\sigma f-\sigma i}{\sigma i}\times 100$$

where *Sw* is the detector sensitivity (%), *σi* represents the initial electrical conductivity (S/m) and *σf* is the final electrical conductivity (S/m).

This configuration was the same when studying the rate of change in the electrical conductivity of a yarn in a resin membrane (*Rr*) and of a yarn in the cracked resin membrane (*Rc*) (Figure 4a, without water). It was also the same configuration for the water detection by absorption on the yarns moulded in a resin and cracked plate (*Sw’*) (Figure 4a). This last test required the membrane sensitivity to permit the imitation of the detection of the water leakage in the membrane.

This was the second principle: The short circuit used the drop of water to create a conductive path between the two parallel yarns (Figure 4b). The electrical signal was detected when the water made the link between the two electrically conductive yarns. To compare these yarns’ properties, the conductance was calculated (Equation (3)) through the resistance measured. This principle was studied on yarns without any structure and on yarns in a resin and cracked plate. To make the test repeatable on all of the yarns, a square of 3 × 3 cm of absorbent paper was added on the parallel yarns to deposit the drop of water. It eliminated the shape of the drop and the problems of absorption that could vary the conductance of the circuit:

(3) $$G\;\left(S\right)=\frac{1}{R}$$

where *G* is the conductance of the circuit (S) and *R* is the resistance of the circuit (Ω).

## 3. Results and Discussion

### 3.1. Preparation of the Samples and Repeatability of the Tests

#### 3.1.1. Resin Influence on the Yarns’ Conductivity

**Figure 5:**
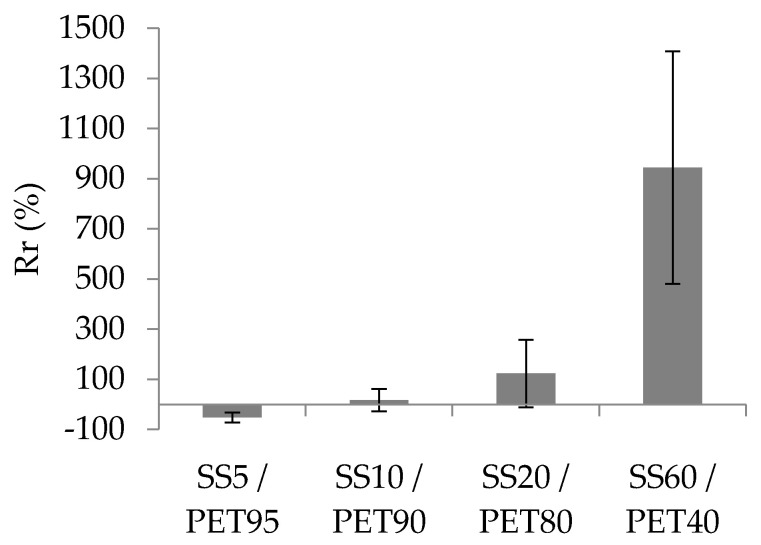
Influence of the implementation of the blended spun yarn in the resin on the yarn’s electrical conductivity.

**Figure 6:**
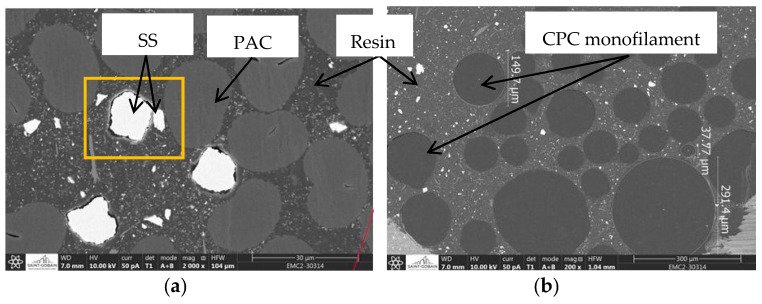
Morphology of the conductive yarns with the resin: (**a**) SS20/PAC80 (the resin compression of the stainless steel (SS) and the polyacrylate (PAC) fibres) and (**b**) PA12 + 3%CNT + 14%CB (separation of the multifilaments by the resin).

The implementation of the yarns in the resin modified the electrical conductivity of the yarns (Figure 5). Here, the rate of change (*Rr*) of the resin was calculated between the initial conductivity of the dry yarn and the final conductivity of the resin yarn. It had two tendencies: a negative rate or a positive rate, meaning that the electrical conductivity after the yarns’ implementation in resin decreases or increases. The SEM images (Figure 6) allowed the formation of some hypotheses regarding these tendencies and the resin’s influence on the fibres.

When the yarns were moulded into the resin, it could modify their conductivity (Figure 5). The *Rr* was higher, with a high proportion of SS in the blend. The *Rr* of the SS5/PET95 was −30%, whereas the *Rr* of the SS60/PET40 was 945%. With a low SS fibre proportion, the resin separated the metallic fibres, inducing a decrease in the electrical conductivity of the yarn. Unlike the separation of the fibres by the resin, the resin compression permitted the reduction of the distance between the SS fibres and thus connected more metallic fibres to each other. This phenomenon increased the electrical conductivity of the yarn in the resin.

This distance reduction hypothesis was more relevant with the SEM images of the SS20/PAC80 yarn (Figure 6a). The *Rr* values for the blend-spun yarns were positive: 209% for SS20/VIS80, 133% for SS20/PAC80 and 123% for SS20/PET80. The distance between the SS fibres, which were white on the SEM image, tended to decrease during the resin implementation of the yarn due to the compression of the resin, the darkest part, on them. This phenomenon of compression was mentioned by Pereira et al. [28], on a yarn of SS/PET in 20/80 proportions by weight, and studied by Tseghai et al. [29], in their work on resistive pressure sensors in stainless steel fabric.

A decrease in the electrical conductivity indicated by a negative rate was noticed for 100% SS-spun yarn, −6%, and CPC yarns such as (PA12)_CNT+CB_, −28%, or PBE60/(PE_KB+CNT_)40, −72%. This phenomenon can be seen on the SEM images (Figure 6b); each monofilament in the CPC yarn, which appears as a grey sphere, was separated by the resin, which appears less dark on the SEM image. When the resin was moulded, it got between all of the CPC monofilaments, which decreased the number of conductive paths of the multifilament.

The resin changed the yarn’s conductivity by compressing or separating the fibres. These phenomena can be observed, but they are not certified due to the high standard deviation of the values. For instance (Figure 5), the error bar of the SS20/PET80 corresponding to the standard deviation of the rates of change was 135%, whereas the *Rr* was 122%. Many of the parameters can modify the yarn’s conductivity, such as the implementation of the yarn in the resin, which is moulded by hand. With this yarn implementation process in the resin, it is difficult to predict the behaviour of the compression or separation of the fibres in the samples.

#### 3.1.2. Cracking Mechanism’s Influence

**Figure 7:**
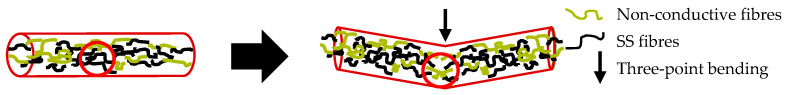
Morphology of the conductive yarns with the cracking process: separation of the SS fibres inside the spun yarn.

The conductivity measurement after the cracking process (Table 5) verified whether the yarn had broken inside the resin during the three-point bending test and showed the behaviour of the yarn’s conductivity after heavy stress (Figure 7).

The rate of change with the cracking process *Rc* (Table 5) was between the initial conductivity of a resin yarn and the final conductivity of the yarn in the cracked resin after the cracking process. It was noticeable that the spun yarn blended with VIS and PAC always broke during the cracking process. On the other hand, the SS5/PET95 and SS10/PET90 yarns did not break during the process, but their conductivity decreased due to their low proportions of SS fibres in the blend. In addition, the SS20/PET80 and SS60/PET40 yarns had negative rates of change *Rc*. This meant the electrical conductivity of the SS/PET decreased after the three-point bending. Hypothetically, this phenomenon can be explained by a sliding of the SS fibres between each other during the cracking process, causing a decrease in the contact points between the conductive fibres and thus a decrease in the yarn’s conductivity. Quadflied et al. [2] also studied the result of a decrease in the electrical conductivity of SS yarns in concrete with a four-point bending process until their breakage in the composite. This can also explain the difference in the *Rc* of the SS20/PET80, which was lower than that of the SS60/PET40. Overall, the more contact points there were between the SS fibres at the beginning, the more likely they were to loosen during the cracking.

### 3.2. Principles of Water Detection

#### 3.2.1. Principle of Absorption

**Figure 8:**
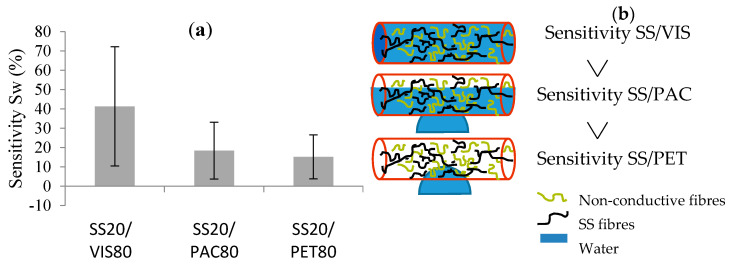
Principle of absorption: influence of hydrophilic property of the non-conductive fibres in the SS-spun yarns: (**a**) detector sensitivity (*Sw*); (**b**) hypothesis of the behaviour of the water-detector yarn.

**Figure 9:**
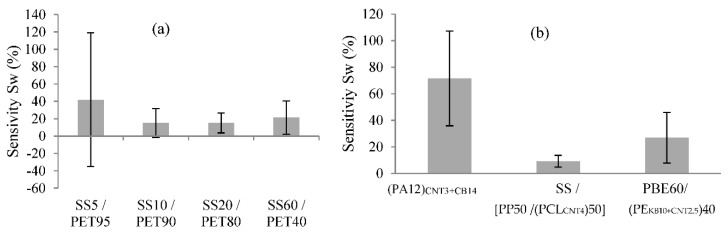
Principle of absorption—properties influence the following: (**a**) proportions by weight of an SS/PET yarn and (**b**) formulation of the CPC.

The water detector sensitivity (*Sw*) based on the variation of the yarn’s conductivity is the rate of change between the electrical conductivity of the dry yarn (initial conductivity) and of the wetted yarn (final conductivity). By comparing the different yarns by the principle of absorption, it was possible to make hypotheses regarding their properties and the detector’s sensitivity. For instance, the sensitivity (*Sw*) tended to increase with the hydrophilicity of the non-conductive fibres (Figure 8). However, the *Sw* decreased with a high proportion of SS in the blend (Figure 9).

The comparison between the non-conductive fibres (PAC, VIS and PET) in the blended spun yarn facilitated linking the *Sw* and the hydrophilic property of the non-conductive fibres (Figure 8a). The SS20/VIS80 has an *Sw* a little bit higher than that of the blends with PAC and with PET, which have hydrophobic properties. The moisture regains of PAC and VIS were approximately the same: 14% at a temperature of 20 °C and a relative humidity of 65%. On the other hand, PET had a moisture regain of 1.5% [30,31]. Therefore, the more hydrophilic the yarn is, the more water the yarn absorbs. The absorbed water creates new conductive paths in the yarn, which increase the *Sw* (Figure 8b). In their work on the development of a conductive ring of spun hybrid yarns in SS/PET or SS/VIS, Shahzad et al. [32,33] studied the effects of the constructional parameters and relative humidity on the electromechanical properties of the yarns. In one part of their study, they investigated the influence of the non-conductive fibres in the blend on the humidity sensors. They observed that normally, the SS/PET yarn was less affected in response to a change in relative humidity (from 44% to 88%) than the SS/VIS yarn, which is more hydrophilic. However, this variation of the electrical conductivity also depended on the proportion by weight of the SS fibres in the blend and the level of the twist factor.

The influence of the proportion by weight of SS was investigated as follows: 5%, 10%, 20% and 60% in the yarns blended with PET were studied (Figure 9a). Notably, the *Sw* was higher with a low proportion of SS in the blend. The drop of water absorbed by the yarns increased the number of conductive paths between the SS fibres. Thus, with a low proportion of SS in the blend, the *Sw* was higher, as in the creation of conductive paths between the water and SS fibres. However, the standard deviation represented by the error bars of certain yarns’ sensitivities was high, especially for the SS5/PET95, which was ±77% (Figure 9a). This indicated that the sensitivity values of the detector with SS-/PET-spun yarns were not steady. The initial conductivities and the structures of the spun yarns were not uniform. This could be due to the formation of a passivation layer on the SS fibre’s surface, which was not homogeneous on all of the fibre surfaces as Yin et al. demonstrated [34]. This phenomenon can create disparities in the values of the yarns’ conductivities, and thus disparities in the values of the *Sw*. The relatively small variation of electrical conductivity between the dry and wetted yarns and the high standard deviations indicated that these types of yarns were inconsistent with the principle of absorption, as concluded by Gaubert et al., who compared SS/PET yarn and silver yarn in water detection [35].

Regarding the 100% SS yarn, the values of the *Sw* did not permit a conclusion regarding the influences of the different yarn structures (spun yarn or multifilament yarn). The *Sw* values of the SS yarns were both less than 1%, confirming the unsuitability of metallic yarns with the principle of absorption. This hypothesis was similar to one developed by Goldfeld et al. [26]. In their study, the variation of the SS yarns’ resistance with the principle of absorption was approximately ΔR = −2 μΩ with distilled water. Therefore, the variation of the electrical conductivity of 100% metallic yarns does not produce a conclusion on the physical structure’s influence (multifilament or spun yarn) on water detection.

The formulation of the CPC yarns—which included various parameters, such as the proportion of fillers, the chemical nature of the polymers in the blend and the physical structure—influenced the *Sw* of the yarn. Comparing the three formulations with different polymers in different proportions (Figure 9b), the *Sw* of the formulation yarn containing the PE, which had a hydrophobic property, displayed the lowest sensitivity. The *Sw* values were steady but depended on the absorption capacity of the yarns and thus on their capillarity, which permitted the spreading of the drop of water along them. Castro et al. [20] studied the effects of different chemical natures of polymer in CPCs on the solvent’s vapour detection. For instance, for the water vapour detection, polycaprolactone (PCL) and the poly(methyl methacrylate) (PMMA) produced the best responses. Thus, these polymeric chemical natures more greatly influenced the water detection.

Regarding the principle of absorption on the yarn in the resin plate, the membrane sensitivity (*Sw’*) was calculated with the variation of the electrical conductivity of a yarn in a cracked resin. This variation is the rate of change between the initial conductivity and the conductivity of the yarn with a drop of water inside the cracked zone. For the SS20/PET80 yarn, the electrical conductivity increased when the drop of water inside the crack touched the yarn: 44 ± 36%. For the SS60/PET40, this principle was not the most suitable because a lot of yarns lose their conductivity after the three-point bending.

#### 3.2.2. Principle of the Short Circuit

**Figure 10:**
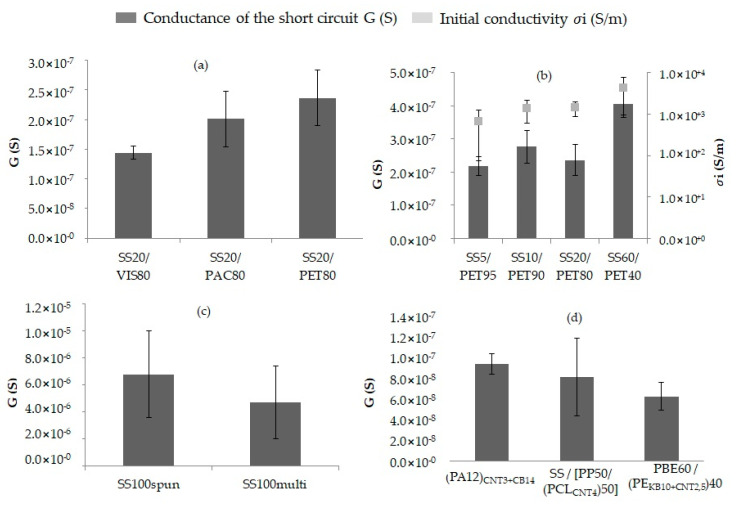
Short circuit: influence of different yarn properties on the signal: (**a**) the chemical nature of the non-conductive fibres in the blend, (**b**) the SS proportion by weight in the SS/PET blend-spun yarns, (**c**) the physical nature of two 100% SS yarns and (**d**) the formulation of different yarns in CPC.

**Figure 11:**
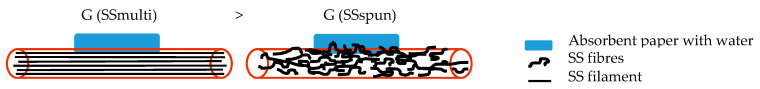
Short circuit: physical structure influence for spun and multifilament yarns.

The short circuit’s signal depended on the configuration of the circuit and the water properties, which were both fixed, as well as on the yarn’s properties. Figure 10 presents the influence of the yarn’s properties on the water detection, such as the physical structure of the SS yarns or the formulation of the CPC yarns. For instance, the conductance tended to increase with the physical structure in the spun yarns (Figure 10c and Figure 11).

The chemical nature of the non-conductive fibres in the blend determined the signal detection speed. The more the non-conductive fibres were hydrophilic, the quicker the water on the absorbent paper was absorbed by the two yarns. The signal after five seconds of stabilization tended to be a little more intensive with the SS20/PET80 yarns due to the PET hydrophobic property. In addition, the number of contact points between the water on the paper and the yarns decreased faster with hydrophilic fibres, such as PAC or VIS (Figure 10a). According to a hypothesis by Martínez-Estrada et al. [36], the non-conductive fibres did not play a significant role in the signal strength.

The conductance of the circuit increased with the SS proportion in the blend (Figure 10b). Therefore, the detection signal was correlated with the initial conductivity (of the dry yarns). The yarn in SS60/PET40 had the highest initial conductivity due to having the highest proportion of SS in the blend, so this detector had the best conductance. The more conductive the yarns were, the better the water detector capacity was.

The physical structure of the SS yarns determined the intensity of the signal (Figure 10c). The water in the middle of the two parallel yarns created more contact points with SS fibres in a spun yarn than in a multifilament (Figure 11). As a result, the conductance of the short circuit was higher with SS-spun yarns: about 2.1 × 10^−6^ S greater than with SS multifilament yarns (approximately 4.7 × 10^−6^ S).

The short circuit’s conductance with the CPC yarns depended on the yarns’ formulation. For instance, comparing the three formulations of CPC yarns (Figure 10d), the one containing the PE, which had a hydrophobic property, had the lowest conductance. Moreover, the conductance values of the short circuit with the CPC yarns were lower than those with the metallic yarns due to having lower initial conductivities: 10^−3^ to 2.5 against 10^2^ to 10^5^ S/m. However, the standard deviation values were lower as well, about 2 × 10^−8^ against approximately 3 × 10^−6^ S for the 100% SS yarns, which were less uniform. Therefore, it was possible to conclude that the electrical signal was more stable.

For water detection with metallic yarns in a cracked resin, the principle of a short circuit is the most suitable. To compare it with the signals without resin, the conductance of the short circuit in the resin plate was lower due to all of the manipulations of the implementation of the yarn in the resin and the creation of the resin crack. For instance (Table 6), the SS20/PET80 had a conductance of 6.5 × 10^−8^ S, and the SS60/PET40 had a conductance of 6.39 × 10^−8^ S. However, even if a yarn broke inside the resin, the signal was ensured due to the creation of electrically conductive paths with the drop of water inside the crack.

## 4. Conclusions

This study examined the influence of the electrically conductive yarns’ properties on water detection and the influence of the detector’s membrane creation and cracking process on their electrical conductivity variations. Two different conductive yarns were used: metallic yarns with stainless steel fibres and CPC yarns with carbon fillers.

First, to observe the influence of the implementation of the detectors’ yarns on the resin and the resin-cracking process, the electrical conductivity variation and SEM images were controlled. The resin, which had a good adhesion with the yarns, modified the conductivity by compressing or separating the fibres. The resin cracking could deteriorate the electric yarns by breaking them or reducing the contact points between the conductive fibres, thus reducing the global conductivity of the metallic yarns.

Next, two principles of detection were examined. Regarding the principle of absorption based on the yarn’s swelling property, certain hypotheses were formed. The detector’s sensitivity increased with the hydrophilic property of the non-conductive fibres and low proportions of the SS fibres in the blend. However, the sensitivity values of the metallic yarns were very low, contrary to their standard deviations; the signal was not steady. Thus, the principle of absorption was more suitable for the CPC yarns. Regarding the principle of the short circuit, the signal is based not only on the capacity of the conductive paths’ creation as the first principle but also on the initial conductivity of the dry yarns composing the circuit. The metallic yarns were more suitable for the principle of the short circuit because they had an electrical conductivity 10^5^ times higher than that of the CPC yarns. Moreover, the structure in the spun yarn created more contact points between the water and the metallic fibres. Therefore, the short circuit principle made it possible to overcome these challenges by ensuring the detection of a fluid leak even if the electrically conductive yarns broke. However, the detection of crack creation is not possible with the principle of the short circuit. Further challenges, such as the influence of the detector’s aging and the repeatability of the detection, need to be faced. Other formulations of CPC yarns will also be the subject of future research to improve the yarn’s capacity for crack creation detection and water detection by the principle of absorption.

## Figures and Tables

**Table 1:** Summary of the different stainless steel blends.

	Physical Structure	Blend Proportion (% by Weight)	Fineness (Tex)	Yarn Conductivity (S/m)	Abbreviation
Stainless steel/Polyacrylate	Spun yarn	20/80	188	8.95 × 10^3^	SS20/PAC80
Stainless steel/Viscose		20/80	185	1.55 × 10^4^	SS20/VIS80
Stainless steel/Polyethylene terephthalate		5/95	203	6.52 × 10^2^	SS5/PET95
Stainless steel/Polyethylene terephthalate		10/90	189	1.37 × 10^3^	SS10/PET90
Stainless steel/Polyethylene terephthalate		20/80	207	1.39 × 10^3^	SS20/PET80
Stainless steel/Polyethylene terephthalate		60/40	196	1.85 × 10^4^	SS60/PET40

**Table 2:** Summary of the different 100% stainless steel yarns.

	Physical Structure	Fineness (Tex)	Yarn Conductivity (S/m)	Abbreviation
Stainless steel	Multifilament	500	7.32 × 10^5^	SS_multi_
Stainless steel	Spun yarn	501	9.45 × 10^5^	SS_spun_

**Table 3:** Summary of the different conductive polymer composite (CPC) yarns.

	Physical Structure	Fineness (Tex)	CPC Conductivity (S/m)	Abbreviation
pa12 + 3%CNT ^1^ + 14%cb ^1^	Multifilament	967	8.49 × 10^−3^	PA12_CNT+CB_
pp/(pcl + 4%CNT) coated stainless steel	Monofilament	601	2.27	SS/(PP50/PCL_CNT_50)
pbe/(pe + 10%kb + 2.5%cnt)	Monofilament	2804	1.57 × 10^−1^	PBE60/PE_KB+CNT_40

^1^ CNT: Carbon nanotubes and CB: Carbon blacks.

**Table 4:** Summary of the various sensitivities measured.

Name	Abbreviation	Initial State of Yarns σi (S/m)	Final State of Yarn σf (S/m)
Water detector sensitivity	*Sw*	Dry yarn (before water droplet deposition)	Wetted yarn (after water droplet deposition)
Rate of change in the yarn’s electrical conductivity in a resin membrane	*Rr*	Dry yarn	Resin yarn
Rate of change in the yarn’s electrical conductivity in a cracked resin membrane	*Rc*	Resin yarn	Cracked resin yarn
Membrane sensitivity	*Sw’*	Cracked resin yarn	Cracked resin yarn after droplet deposition

**Table 5:** Cracking mechanism influence on the electrical conductivity of the SS20/PET80 and SS60/PET40 yarns.

	Rate of Change Rc (%)	Standard Deviation (%)
SS20/PET80	−18.09	36
SS60/PET40	−68.73	26

**Table 6:** Principle of the short circuit of the SS20/PET80 and SS60/PET40 yarns in the resin.

	Conductance of the Yarn in the Resin G (S)	Standard Deviation (S)
SS20/PET80	6.5 × 10^−8^	9.65 × 10^−9^
SS60/PET40	6.39 × 10^−8^	1.06 × 10^−8^
